# The Eight and a Half Year Journey of Undiagnosed AD: Gene Sequencing and Funding of Advanced Genetic Testing Has Led to Hope and New Beginnings

**DOI:** 10.3389/fendo.2017.00107

**Published:** 2017-05-19

**Authors:** Illana Gozes, Marc C. Patterson, Anke Van Dijck, R. Frank Kooy, Joseph N. Peeden, Jacob A. Eichenberger, Angela Zawacki-Downing, Sandra Bedrosian-Sermone

**Affiliations:** ^1^The Lily and Avraham Gildor Chair for the Investigation of Growth Factors, Elton Laboratory for Neuroendocrinology, Department of Human Molecular Genetics and Biochemistry, Sackler Faculty of Medicine, Adams Super Center for Brain Studies and Sagol School for Neuroscience, Tel Aviv University, Tel Aviv, Israel; ^2^Division of Child and Adolescent Neurology, Pediatrics and Medical Genetics, Mayo Clinic Children’s Center Rochester, Rochester, MN, USA; ^3^Cognitive Genetics Group, Department of Medical Genetics, University of Antwerp, Antwerp, Belgium; ^4^Diagnostic Clinic, East Tennessee Children’s Hospital and Clinical Assistant Professor of Medicine at the University of Tennessee, Knoxville, TN, USA; ^5^Physician Informaticist, Children’s Hospital of Georgia at Augusta University, Augusta, GA, USA; ^6^ADNP Kids Research Foundation, Brush Prairie, WA, USA

**Keywords:** activity-dependent neuroprotective protein, case study, mutation, nonsense, motor delays, autism spectrum disorder

## Abstract

**Background:**

Activity-dependent neuroprotective protein (ADNP) is one of the most prevalent *de novo* mutated genes in syndromic autism spectrum disorders, driving a general interest in the gene and the syndrome.

**Aim:**

The aim of this study was to provide a detailed developmental case study of ADNP p.Tyr719* mutation toward improvements in (1) diagnostic procedures, (2) phenotypic scope, and (3) interventions.

**Methods:**

Longitudinal clinical and parental reports.

**Results:**

AD (currently 11-year-old) had several rare congenital anomalies including imperforate anus that was surgically repaired at 2 days of age. Her findings were craniofacial asymmetries, global developmental delay, autistic behaviors (loss of smile and inability to make eye contact at the age of 15 months), and slow thriving as she gradually matures. Comprehensive diagnostic procedures at 3 years resulted in no definitive diagnosis. With parental persistence, AD began walking at 3.5 years (skipping crawling). At the age of 8.5 years, AD was subjected to whole exome sequencing, compared to the parents and diagnosed as carrying an ADNP p.Tyr719* mutation, a causal recurring mutation in ADNP (currently ~17/80 worldwide). Brain magnetic resonance imaging demonstrated mild generalized cerebral volume loss with reduced posterior white matter. AD is non-verbal, communicating with signs and word approximations. She continues to make slow but forward developmental progress, and her case teaches newly diagnosed children within the ADNP Kids Research Foundation.

**Conclusion:**

This case study emphasizes the importance of diagnosis and describes, for the first time, early motor intervention therapies. Detailed developmental profile of selected cases leads to better treatments.

## Introduction

Looking at neuroglial interactions, we (Gozes group) discovered activity-dependent neuroprotective protein (ADNP), as a protein secreted from glial cells in the presence of vasoactive intestinal peptide (VIP), which mediates VIP’s neuroprotective activity (1). Further cloning (2) revealed high conservation and specificity to vertebrates (3). The large human ADNP (hADNP) gene structure (~40 kb) includes five exons and four introns with alternative splicing of an untranslated second exon (chromosome 20q12-13.2, a region associated with aggressive tumor growth). As we described (2, 3), hADNP is also mutated in cancer.

Knocking out ADNP in mice resulted in embryonic death at the time of neural tube closure, revealing that ADNP is crucial for brain formation (4). ADNP haploinsufficient mice survive but show learning and memory deficits (5), in a sex-dependent manner (6). At the protein level, we have shown multiple crucial interactions for ADNP including direct binding to the chromatin remodeling complex SWI/SNF and interaction with heterochromatin protein 1 alpha (7, 8). This study was extended to show that ADNP interacts with all HP1 proteins toward histone posttranslational modification (9). At the transcriptional level, ADNP binds to the locus control region of the beta globin gene to regulate globin transcription (10) as well as the promoter regions of apolipoprotein E, cathepsin C, cathepsin Z, metallothionein 1, neurogenin 1, and myosin regulatory light chain 2 (8). At the RNA splicing level (11), ADNP interacts with Brahma, a component of the SWI/SNF complex regulating alternative splicing that shows a similar developmental expression pattern to ADNP. Immunoprecipitations further suggested binding between ADNP and polypyrimidine tract-binding protein-associated splicing factor (PSF), with PSF being a direct regulator of the microtubule-associated tau transcript splicing. Further interaction with the protein translation machinery was shown with the eukaryotic translation initiation factor 4E (eIF4E) (6) through direct binding as well as sex and age-dependent regulation. Notably, eIF4E is linked to autism (12). In the neuronal cell cytoplasm, ADNP is critical for neurite outgrowth and maintenance (13) through direct interaction by its SIP motif with microtubule end binding proteins (14, 15). The microtubule system is tightly associated with the autophagy system (16), and ADNP binds the microtubule-associated protein 1 light chain 3B (17). With these key regulatory functions ADNP controls the expression of >400 genes during embryonic development (8) and of thousands of hippocampal genes postnatally, impacting pathways associated with ion channels-synaptic transmission in a sex- and age-dependent manner (18).

From a clinical point of view, an ADNP gene deletion was first implicated in delayed cognitive development in a case study in 2007 (19). Further findings included the first *de novo* p.Lys408Valfs*31 mutation in the ADNP gene in a large cohort of autistic patients (20). Large-scale sequencing study (21), analyzing 2,446 probands, identified an additional p.Tyr719* *de novo* ADNP mutation and this is the subject of our current publication. Helsmoortel et al. (22) grouped 10 patients with mutations in ADNP. As all patients suffered from autism with intellectual deficiencies and shared characteristic facial features, it was concluded that mutations in ADNP cause a syndromic form of autism. Two additional patients were further described, sharing the reported characteristics (23, 24). Interestingly, Pescosolido et al. described another case of p.Tyr719* *de novo* ADNP mutation, suggesting that this is a recurrent mutation. Coe et al. (25) also reported five patients with a truncating ADNP mutation in a screening of 4,716 patients with autism/ID. De Rubeis et al. (26) identified three more patients of a total of 3,871 screened and the DDD project reported four novel cases out of 1,133 screened (27). We have summarized the above in an introductory remark to the story of Tony Sermone, presenting an additional ADNP mutation L349Rfs*49 (28). A short synopsis of the clinical phenotype was recently published (29), additional mutations are discovered and information extended (27, 30–33) and an extensive description is in preparation (Van Dijck et al., in preparation). Interestingly, a recent publication suggested that ADNP is one of three major genes associated with autism spectrum disorder (ASD) (34), complementing the original estimation of the ADNP-related syndrome constituting 0.17% of ASD cases (22). These findings mark ADNP as one of the most frequent ASD-associated genes known to date. Therefore, further understanding of the ADNP-related syndrome is of general interest both from a case study point of view as well as from a population perspective. Here, we chose to present one case study and concentrate, for the first time, on delays in motor development.

## Methods

Longitudinal clinical and parental reports were collected focusing on diagnosis, phenotypic scope, and interventions. Time points of emphasis: birth, 1–3.5 years and 8.5 to date. All materials were given with parental informed consent.

## Results

### Initial Clinical Data

Father’s and mother’s age at birth was 32 and 31, respectively. No consanguinity. No affected siblings—parents were carriers of CF. AD was born at 37–38 weeks vaginally (October 1, 2005). She was 50 cm in length and weighed 3.475 kg. Her head circumference was 34 cm. She had Apgar scores of 7 and 8 at 1 and 5 min of life. She was noticed to have low anal atresia and had surgery to repair this at 2 days of age [Table 1 (A), physical anomalies]. She had significant GERD as an infant and young child. At 2 years of age, AD was diagnosed with autism.

**Table 1:** **Parental/caregiver observations/clinician observations**.

**A: Gross physical anomalies**											
Age (years)	0 Imperforate anus (surgery at 2 days, 12-day stay)—5 months anal dilation	1	2	3	4	5	6	7	8–10		
Legs	Extreme small feet and toes. At 9 years and 8 months: trunk and upper extremities appear larger than lower extremities. Left leg is 2 cm shorter than the right leg and 9 cm less in width at the midthigh. Her left foot is smaller than her right. She has fifth finger clinodactyly. She has proximal implantation of the thumbs										
Hair	White/blonde (silver) forelock of hair (remaining hair is brown), hair growth pattern discrepancy (left side). Low hairline. She has a posterior parietal hair whorl. She has an irregular hair part to the left										
Face	Facial asymmetry, flat face and a slant mouth (left side), small low-set ears posteriorly rotated, facial palsy (age 15 months–8.5 years of age)										
Head and face	Flat back/head side (plagiocephaly). Treated with helmet cranial technologies. Frequent otitis as an infant/young child	Stopped smiling, had trouble with eye contact (~15 months)				Stopped head helmet treatment, suggested microcephaly			Eye defects: she has intermittent left esotropia (a right gaze preference). Prominent upper buccal frenulum. Widely spaced and asymmetric size of teeth. Mandibular dimple upon her chin that is to the left of the midline		
Stature	60th percentile (%)							10%	“Short” stature	0% short stature	
Weight						48–50%					
**B: Motor disabilities and sleep disturbances**											
Age (years)	0	1	2	3	4	5	6	7	8	9	10
Legs	Leg length discrepancy (left leg), extreme small feet and toes, low tone, hypotonic throughout the body										
	Left side weakness “abnormal gait” favoring right side										
	Legs showing signs of atrophy–not growing until 2.5 years				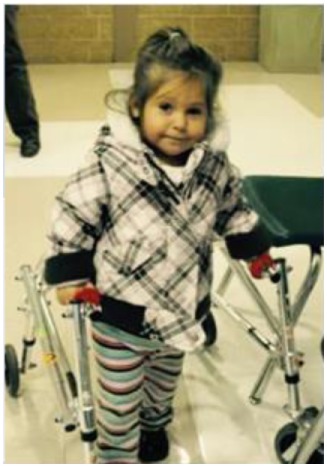			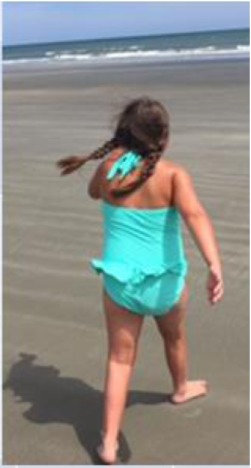		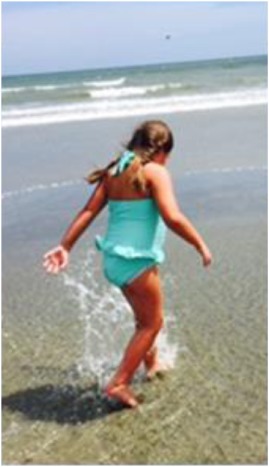	
Muscles	Extreme muscle tightness (warm baths, stretching, braces, treadmill, and orthopedic shoes, >300 treatments)										
Mobility	Sitting up at 9 months and severe delays starting at 11 months. Minimal progress	Most severe motor delay minimal progress—baring weight to stand was severely delayed until 3 years of age									
Walking		Therapy began at 15 months		Begin independent walking (3.5)							Running!
Fine muscle tone	Made improvements, still struggling										
Sleep disturbance	Until 3 years of age, terrible spells (stopped breathing) required ambulatory care/inpatient hospital visits. Apnea tests revealed nothing. Nightly awakening l–4 a.m., muscle cramps and dystonia, until the age of 5 years of age							Improved after 5		Normal sleep	Hypotonia
**C: Clinical tests**											
**Karyotype (skin biopsy):**						(46,XX)—no chromosome abnormality was apparent.					
**Array Comparative Genomic Hybridization**						arr cgh 1-22(39,986 oligos)x2,X(2,745 oligos)x2,Y(367 oligos)x0. The result is normal. No abnormality was detected by array CGH analysis					
**Prader–Willi–Angelman analysis**						MLPA demonstrated a normal methylation pattern. No deletions or duplications were detected. This indicates that both the maternally and paternally derived copies of the PW/AS critical region are present					
**Fragile X analysis**						CGG repeat: 23 and 29; methylation status: normal; final result: normal					
**CFTR gene, full gene analysis**						A mutation was NOT detected. Intron 8 poly T alleles are: 7T/7T					
**Endocrine studies** (for growth deceleration) (2 years, 5 months): the sedimentation rate was normal. Calcium was normal at 9.7, and the phosphorus was normal as well at 4.7 mg/dL Reference range for the calcium is 9.6–10.6, and the reference range for the phosphorus is 4.3–5.4. Normal thyroid function: TSH was 2.7 with a reference range of 0.3 to 5, and the free T4 was 1.3 with a reference range of 0.8–1.8						Growth factors were also in the normal range. Specifically, the IGF-1 was 54 ng/mL The reference range is 51–303. The IGFBP-3 is at 3.7 mcg/mL with a normal range being 0.8–3.9. The skeletal survey showed bone age at 2.5 years using the RU5 method; there was no evidence of skeletal dysplasia					
**Echocardiogram** (2 years, 5 months)						Normal					
**Upper Gl endoscopy and flexible sigmoidoscopy** (2 years, 5 months)						The upper endoscopy was completely normal on both visualization and histology. During the flexible sigmoidoscopy, the presence of a significant amount of stool was noted, and AD was disimpacted					
**Retroperitoneal ultrasound** (2 years, 5 months): **additional evaluation at 9 years, 8 months:** delayed bladder training						Both kidneys are in normal position and have normal appearance. No hydronephrosis or parenchymal loss. The right kidney measures 6.8 cm; left, 6.9 cm. These are normal measurements for a patient of this age. Ureters are not seen. Incidentally noted is an enlarged spleen with several calcified granulomas within it. The spleen measures 9.6 cm					
**D: Psychology performed at 3 years, 2 months**											
**Bayley Scales of Infant Development, Third Edition:** AD was able to attend to a picture, squeeze object to make a sound, retrieve an object of interest, stack two blocks, scribble on paper with crayon, make forward progress by crawling, and make coordinated, alternating step movements (language assessment was performed separately by Speech Pathology)											
**Autism Diagnostic Observation Schedule:** AD had limited language, including three signs (more, all-done, and bubbles) and a few verbalizations (babbling, whining, groan, and words “go,” “da-da,” and “ma-ma”). She did not have a distal point. She had poor eye contact; rarely or never directed facial expressions at others; and did not coordinate eye contact, verbalizations, and gestures to communicate social intent. She occasionally moved closer to person as a request for more or would moan indirectly when frustrated. She did not respond to name, except when her father implied he was going to tickle her. She signed “more” for bubbles and snacks. She was generally aloof and passive and did not exhibit giving, showing, or initiate joint attention. She responded to joint attention when toy was activated but not in response to gaze or pointing by examiner. She played with cause and effect toys and tended to perseverate on musical phone toy. She exhibited many self-stimulatory, repetitive behaviors including: (a) holding her hands out in front of her and waving her hand while staring and smiling at it, (b) slapping her belly hard then holding her hand up in the air and shaking and staring at it while laughing, and (c) putting her head back, shaking her hair, and staring at the lights. She also peered at objects out of the corner of her eyes, banged her head on floor, and exhibited rigid body and flapping hands when excited about bubbles											
**Clinical report at 9 years and 8 months**											
**Has several autistic behaviors:** swishing of her head and hair back and forth. Head banging. She looks at objects out of the corner of her eyes in a stereotypical fashion. She has inconsistent response to her name (despite normal hearing). Her play behavior is largely perseverative play with basic toys. She enjoys music. She does not have pretend play. She will clap her feet together and stomp when excited. She clinches her hands together with excitement. She can be friendly and loving; however, she may also have aggressive periods											
**E: Evaluation by Speech Pathology at 3 years, 2 months**											
**The Verbal Language Development Scale was administered:** this is a developmental scale that is completed *via* observation of the child as well as through parent report. AD exhibited a language age equivalent of 12 months on this instrument. Those items which she exhibited that would be expected by the first year of age include laughing, smiling, producing consonant sounds reflexively, understanding the word “bye-bye”, and following simple directions. She was inconsistently responding to her name. She did not yet imitate sounds or imitate words. Those items that she exhibited that would be expected between the first and second year of age include an expressive vocabulary of two words and recognizing the name of familiar objects; marking with a pencil or crayon was emerging. Items that would be expected by the second year of age that she did not yet exhibit included an expressive vocabulary of at least 25 words, using the names of familiar objects, identifying common pictures when they are named, talking in short sentences, and naming common pictures											
**Expressive language:** a spontaneous language sample was taken throughout this session. Much of this was done while AD was playing with toys or interacting with her parents or interacting with her sister. A number of instances of non-verbal communication including nodding yes to a question and using a sign to indicate that her parents were being bad were observed. A number of isolated syllables using the neutral schwa vowel and /m/ and /n/ were heard. As well as one instance of “da”. She also used the vowel /i/ in isolation in a non-meaningful way. By parent report, she also produced a repetitive “dibi, dibi, dibi.” Therefore, her phonetic inventory is constrained to /n/, /m/, /d/, /b/, and /p/. Vowels were constrained to primarily the schwa, /I/, and an isolated /i/. Mr. and Mrs. parents report they have not heard additional vowels at home. Syllable shapes noted included consonant–vowel and reduplicated syllables											
**Oral structural functional exam:** physical examination of the oral mechanism was done *via* observing AD during vocal play. She had facial symmetry at rest. Range of motion of the lips is full bilaterally for retraction and for lip rounding. Tongue movement was not observed. Given her good production of “da,” she shows good strength of lingual movement for the plosive sound. She also is reported to produce /b/ and /p/ showing good labial strength. There did not appear to be any reduction in range of motion. The presence of oral non-verbal apraxia could not be ascertained as she was not yet able to follow verbal directions or imitate blowing, clicking her tongue, puckering lips on command, etc. (This was diagnosed later at ~10 years of age)											
**Speech production:** spontaneous speech is characterized by adequate respiratory support and a mildly hoarse phonatory quality (+1). Resonance is normal. There was no evidence for dysarthria											
**Behavioral and communication observations:** AD showed fleeting eye contact. She played appropriately with a number of toys. She showed good attention to her sister’s face as her sister engaged her in attempts at imitation of sounds. She sought attention both from her parents and from the clinician. A couple of spontaneous signs were noticed. She did babble sounds as noted above but less frequently than would be expected even for a child 9–12 months of age. There were some moments of joint attention but this was inconsistent. Her parents reported frequent head banging; however, this was not observed during the clinical session											
**F: Behavior dysfunctions**											
Age (years)	0	1	2	3	4	5	6	7	8	9	10
Autistic-like		15 months (lack of smile)	Non-verbal, began head banging (self-injury)	3.5 began walking, smile develops	Much injurious behavior disappeared (reduced) as she started walking, communication cards and voice technologies				Some injurious behavior		Non-verbal, being trained and can sign and use voice technology, increased vocalization and independency. Receptive language grew with aggressive repetition. Her participation increased with adaption of her needs. Anxiety is subsiding
Treatment benefits			Greatly benefited from developmental playgroups prior to age 3.	Parental observation: AD is known to smile and any display positive body language toward normally developing peers (she smiles and even waves hi and tries to approximate goodbye “beh” to other children and now as she ages she no longer needs prompting for the social interactions). Multiple therapies are the best treatment. She benefited and grew in independence both in mobility and functionality with the public school system							
Sensory		Dysfunction disorder diagnosed prior to the age of 2. Difficulties with maintaining temperature, keeping on gloves, hats, shoes, etc.									

*(A) Gross physical anomalies and sleep disturbances. (B) Motor disabilities (including pictures at toddler age with walker and running at the age of 10). Findings are arranged by age (years) 1–10. (C)–(E) Clinical observation: AD was seen in multidisciplinary consultation at Mayo Clinic in 2008, at 3 years of age. The multidisciplinary team at that time included Pediatric Neurology, Pediatric Endocrinology, Physical Medicine and Rehabilitation, Medical Genetics, Social Work, Pediatric Allergy, Pediatric Cardiology, Pediatric Gastroenterology, Pediatric Otorhinolaryngology, Pediatric Dermatology, Orthopedics, Developmental and Behavioral Pediatrics, and Neuropsychology. (C) Clinical tests. (D) Psychology performed at 3 years, 2 months. (E) Evaluation by speech pathology at 3 years, 2 months. (F) Behavioral dysfunctions (findings are arranged by age in years: 1–10)*.

### Developmental Delay: Focus on Motor Function

AD started sitting at 7–9 months. While her younger brother walked at 1 year of age, she continued to lag severely behind, she could not crawl, walk, or even bare weight to stand on her legs. It seemed that at about 2 years of age, AD’s legs began to show some signs of atrophy, not increasing in shoe size (unlike her siblings), until she began to walk. Currently, at the age of 11, one can still see the disproportion of AD’s small tapered legs from the knees to the feet, compared to the rest of her body. This finding attests to the importance of ADNP in muscle and bone development and to the regulation of key gene/proteins beyond embryonic development (8, 18).

**Figure 1:**
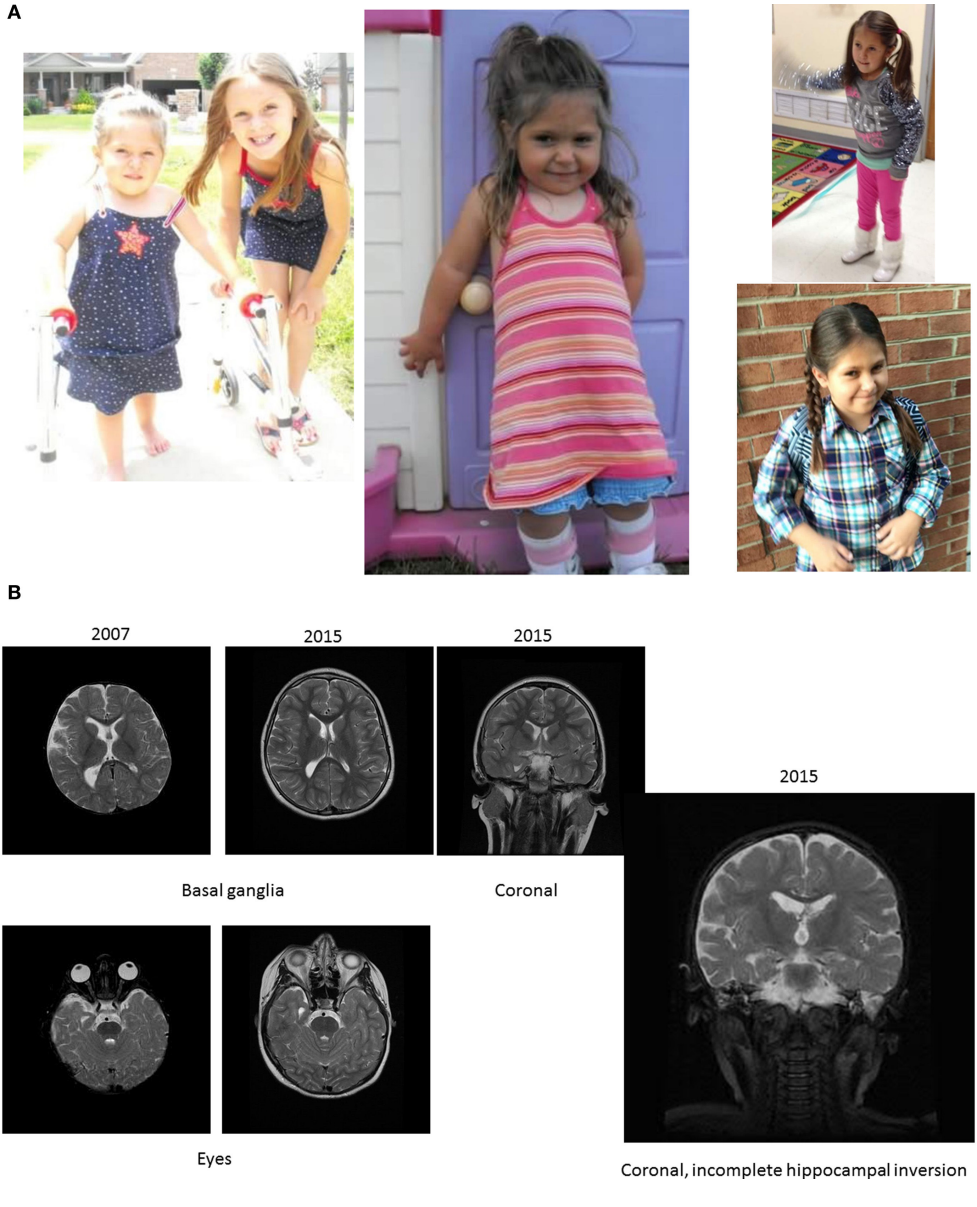

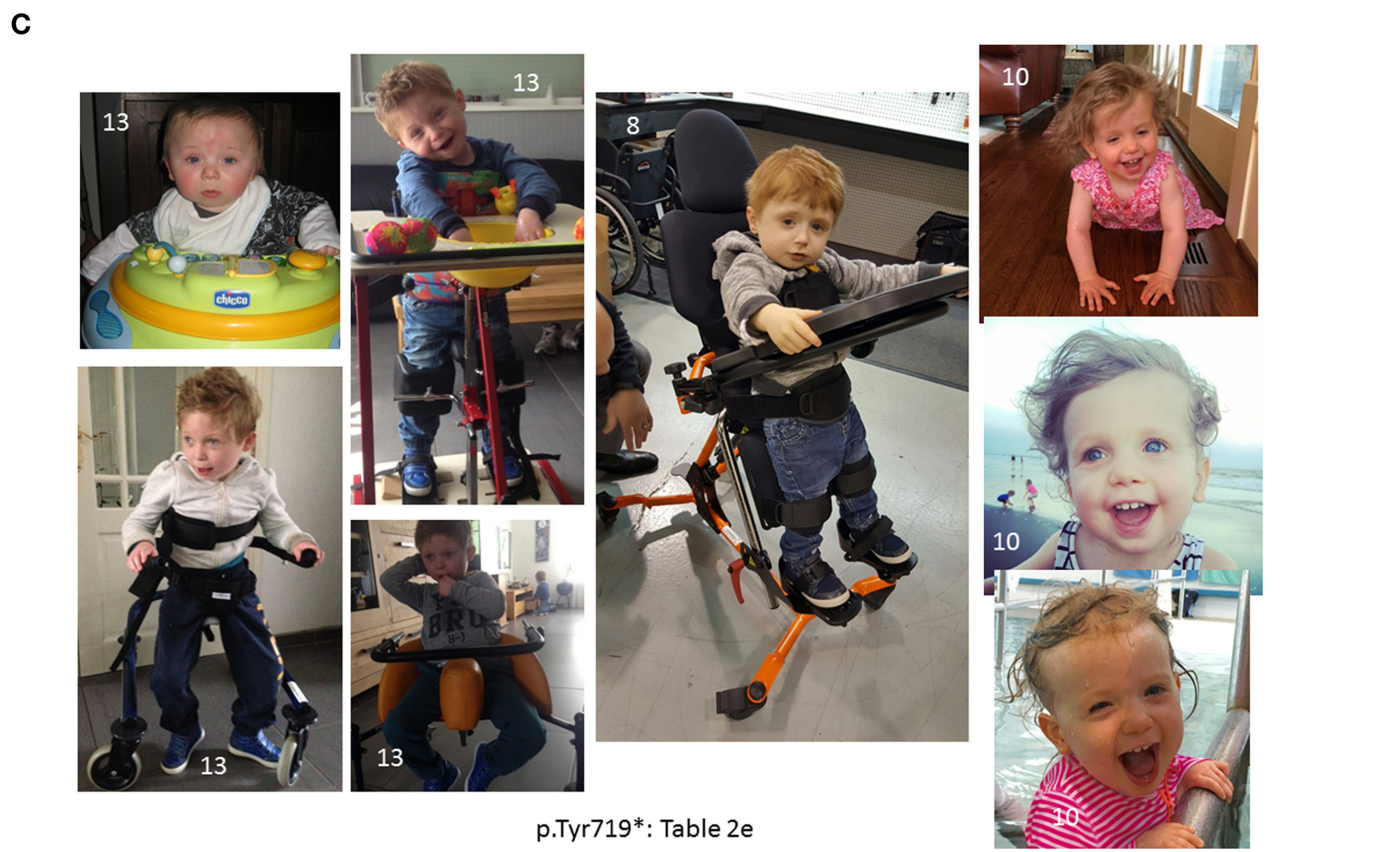
**Pictures depicting AD’s development, brain development, and comparison to other children with the same mutation**. **(A)** AD’s development in pictures: starting top left: young AD with her sister’s “push” to walk. AD is barefoot and her legs turned red from a very short walk. Her muscles in her ankles got tight and she exhibited low muscle tone and needed to be stretched several times a day, or she would suffer excruciating pain at night. The painful day walk and nights were full of crying periods. At the same time, a picture was taken, when she finally could lean against things with her leg braces and at a similar time point. Third picture on the top, AD at her first beauty pageant, after months of her sister and mother teaching her how to spin and dance with a baton ribbon. Fourth picture on the bottom, AD at her eighth birthday, just before her diagnosis and submission to brain scans. AD is successful at attending regular primary schools. Her smile keeps the family and caregivers going. **(B)** Magnetic resonance imaging results (volumetric T2 performed at 2007 and 2015). **(C)** Pictures of additional children with ADNP p.Tyr719* mutation (featured also in Table 2 (E), all materials given with parental informed consent).

AD started walking with a walker at 2.5 years of age, skipping crawling [Table 1 (B); Figure 1A]. Walking short distances resulted in skin redness and muscle pain. She began walking independently at the age of 3.5 years. The following year presented daily challenges in her “functional” walking. She often could not stand for more than a few seconds. Climbing stairs was a third milestone, using the strong leg and pushing the weaker leg after. Until about the age of 5, AD had severe muscle tightness that mimicked symptoms of a neuromuscular disorder. The muscle tightness was associated with severe sleep disturbances [Table 1 (B), last raw]. At 9 years and 3 months, the parents noticed that she had more of a delay with gross motor skills than with fine (albeit both delayed). She walked independently, but with a broad based and antalgic gait [Table 1 (B); Figure 1A]. At 10.5 years of age, she was ambulatory and quite flexible with increased range of motion noted particularly in the small joints. Physically, hands were broad, with short and broad fingers and fifth finger clinodactyly. Distal phalanges appeared short and fingernails were spatulate. Feet showed short fourth and fifth toes and deep setting. Toe tip and fingertip pads were prominent [see also Table 1 (A)]. Through parental persistence and AD’s willpower (see treatments below), now at age 11, she is running and riding a bicycle. She still struggles with reciprocating down stairs and is mostly non-verbal (see below).

### Clinical Test Results and Growth Curve

#### Age of 3 Years and 2 Months: (Mayo Clinic)

1. Static encephalopathy of presumed antenatal onset and genetic etiology
2. Severe global developmental delay (the diagnosis of intellectual disability is typically not made at this age)
3. ASD
4. Multiple congenital anomalies (imperforate anus, dental anomalies, facial asymmetry, and poliosis)
5. Microcephaly [head circumference, 45.5 cm (<2%)]
6. Generalized hypotonia, axial greater than appendicular
7. Normal stature and mass [weight 14.7 kg (61%); length, 90 cm (10%)]
8. Hyperopic astigmatism

Extensive investigations showed normal or negative results [Table 1 (C)], those also included tests for Rett syndrome and a skeletal survey (bone, age 2.5 years). Psychological and speech evaluations were also included showing global delays as indicated below.

#### At 9 Years and 3 Months

AD’s weight was 27.1 kg. Her height 114.7 cm, occipitofrontal circumference 50 cm. She has continued problems with constipation/obstipation.

#### At 10.5 Years of Age

She was short statured (height 128 cm)^1^ with normal weight and head circumference (34.5 kg and 52 cm, respectively). She was still mostly non-verbal. She communicated with her family with signs and word approximations. She demonstrated autistic stereotypies as well as sensory-seeking behaviors including head banging and skin picking.

Currently, AD continues to make slow but forward developmental progress, demonstrating the importance of early and extensive therapies (see below).

### Psychological Test Results (3 Years and 2 Months, Mayo Clinic)

Bayley Scales of Infant Development, Third Edition: (SS = 55, first percentile) 11-month-old level [Table 1 (D)]. Motor skills (SS = 49, <0.1st percentile) 15-month-old level for fine motor skills to 9-month-old level for gross motor skills.

Child Development Inventory (MCDI): general development 16-month-old level, social maturity, 23-month-old level; self-help skills, 15-month-old level; fine motor and language skills, 16-month-old level; and gross motor skills 11–12 months.

Behavior Assessment System for Children, Second Edition–Parent and Teacher Rating Scales: “at risk” for attention problems and sad/irritable mood.

Conners Teachers Rating Scale, Revised: limited adaptive skills, including socialization and functional communication.

School Situation Questionnaire: AD’s behavior was not reported to be problematic in most school situations.

Autism Diagnostic Observation Schedule: communication (RS = 4, cutoffs = 2/4), reciprocal social interaction (RS = 14, cutoffs = 4/7), combined algorithm (RS = 18, cutoffs = 7/12); limited play skills (RS = 4) and stereotyped behavior and restricted interests (RS = 5). Consistent with ASD [Table 1 (D)].

### Evaluation by Speech Pathology (3 Years and 2 Months)

The Verbal Language Development Scale: age equivalent of 12 months.

Expressive language: a number of instances of non-verbal communication observed and a number of isolated syllables; no recognizable words.

Oral structural functional exam: facial symmetry at rest; range of motion of the lips was full bilaterally for retraction and for lip rounding; reported adequate tongue movement and labial strength. No reduction in range of motion.

Speech production: adequate respiratory support and a mildly hoarse phonatory quality (+1). Resonance is normal. No evidence for dysarthria.

Behavioral and communication observations: fleeting eye contact; played appropriately with a number of toys; good attention to her sister; sought attention both from her parents and from examiner. Babbled sounds less frequently than would be expected even for a child of 9–12 months. Inconsistent joint attention. At ~10 years of age, verbal apraxia was diagnosed [Table 1 (E)].

### Brain Magnetic Resonance Imaging (MRI) Results: Mild Generalized Cerebral Volume Loss with Reduced Posterior White Matter at 15.5 Months and at 9 Years

Scans demonstrated mild generalized cerebral volume loss with reduced posterior white matter. The 2007 study showed periatrial signal hyperintensity that had resolved by 2015. The corpus callosum appeared relatively proportional to the overall volume loss but slightly foreshortened, consistent with brachycephaly. The right lateral ventricle was more asymmetric than normal and much of that can be attributed to the incompletely inverted hippocampus on that side with a low-lying fornix. The frontal and temporal extra-axial spaces were enlarged in 2007, but had resolved by 2015. Craniofacial dysmorphism (prominent forehead, broad nose, and thin upper lip) was apparent on the 2015 scan [Table 1 (A); Figure 1B]. Similarly, at 10.5 years clinical observations, craniofacies showed tall forehead with high anterior hairline, heavy brows giving eyes a deep-set appearance, right exotropia, and long eyelashes. Philtrum was short and deep, and the upper lip was thin. There were creases on the earlobes.

### Molecular Test Results: Part of a Growing Syndrome

Primary diagnosis (Dr. Peeden) was obtained at 8.5 years showing ADNP *de novo* gene mutation: g.49509094G>C; cDNA: c.2157C>G; protein: p.Tyr719*. Diagnosis was obtained by whole exome sequencing compared to the parents. With the ADNP kids belonging to a family of affected children suffering many similar abnormalities, further solutions are on the horizon. To provide information, advocacy and emotional support to families worldwide [e.g., Ref. (28)], an ADNP Syndrome Parents Group was established on the social networking site Facebook™. This is facing the time, like other support groups, for versatile diseases (35). Currently ~80 families are assembled, connected through the original set up of ADNP ASD mutations^2^. The parents formed a table of traits^3^, which are shown for AD [Table 2 (A–D)]. Several other ADNP-associated traits are visible when looking at Table 2 including auditory impediments, suggesting an involvement of ADNP in the development of all senses. AD was spared heart abnormalities and seizures; however, as these affect other ADNP children, she is closely observed (28).

**Table 2:** Activity-dependent neuroprotective protein (ADNP) phenotype data questionnaire (Facebook™, A–D); (E) selected children with ADNP p.Tyr719* mutation (featured also in Figure 1C); Facebook data.

**A**			
cDNA/nucleotide		c.2157C>G	
Protein—pchange		p.Y719	
Zygosity		Heterozygous	
Inherited/*de novo*		*De novo*	
Location		USA	
Sex		Female	
Birthday		01/10/2005	
Pregnancy or birth complications?		Meconium stained from AD imperforate anus	
Birth weight		7.8 lbs	
**Developmental**		**–**	
Gross motor delay		Yes	
Intellectual disability		Originally diagnosed as severe—at the age of 10 diagnosed as moderate	
Speech delay (has under 25 understandable words)		Apraxia/speech delay	
Have you done prompt, rest, or other “oral motor planning” types of therapy or “general speech therapy”		Yes	
Can your child write?		Age 10: now writes her name with hand over hand assistance and used handwriting with no tears program. Primarily a “motor planning issue” needs repetition and tracing. She is also able at age 10 to recognize certain site words on Flashcards especially written in red which helps her cortical vision impairment (CVI)	
Can your child feed him/herself with utensils?		Yes, with spoon	
**Neurological/brain**		**–**	
Has your child had a magnetic resonance imaging (MRI) brain scan?		At 2 years (no findings) and at 9 years (Figure 1A)	
Does your child have a brain abnormality?		Slight diminishing of white matter in the cerebellum (9 years of age)	
Regression of skill?		Slight when not working consistently	
Facial palsy/bells palsy?		Left side facial palsy	
**B**			
**Behavior**		**–**	
Autism spectrum disorder (ASD) diagnosis?		At 2 years of age	
Did you have a hard time getting an ASD diagnosis?		No—however, socializes with adults important: at 3.5 years of age when she achieved stages of independent and functional walking and attained mobility, she started opening up and was able to access her environment and peers. She grows and flourishes (and still does at age 10) having “inclusion” and access to her normally developing peers. AD “wants” to play but her lack of speech makes connecting difficult on many levels.	
Rocks back and forth or shakes head when excited		No (claps her hands and makes noise or word approximation)	
Like to rub or twirl fingers and hands, sometimes close to face for stimulation?		Yes	
Does/did your child love to be around most adults, not just family (loving, eye contact, happy with adults, especially as an infant/young toddler)?		Yes	
Does your child interact or directly socialize with other children?		No (starting to develop as she attained mobility)	
Does your child like “indirect” interaction with children? (prefers to play alone by other children, but not with them)		Yes	
Does/did your child have an extremely loving, friendly, affectionate/cuddles demeanor as a baby/young toddler?		Yes	
Does your child line up or stack items compulsively?		No	
Does your child have an extreme love for music?		Yes!!	
Does your child have an extreme love for water (in water or splashing/playing with water)?		Yes	
Sensory processing disorder?		Yes	
Enjoys/loves “swinging” = vestibular stimulation		Yes	
ADHD diagnosis?		No	
OCD diagnosis?		No	
Anxiety disorder?		No	
BAD Behavior, does/did your child have increased BB in early childhood?		Yes	
What age did “bad” behaviors begin (symptoms)		2 (head banging, hair pulling, copying with waiting, violent behavior—improvements as she matures)	
**C**			
**Vision**		**–**	
Vision impairment		Yes	
CVI		Yes	
Farsightedness		Yes (farsighted for many years—however, with further maturation, this is diminished and now she is tending toward near sightedness)	
Near sightedness		Developed to some degree with further maturation (age 10)	
Astigmatism		Yes	
Glasses?		Transient	
Will your child wear the glasses?		No	
Light-gazer (stared/stares at lights)		Yes	
Strabismus (occasional eye crossing)		Yes	
Ptosis (drooping or falling eyelid)		No	
**Hearing**		**–**	
Developmental hearing impairment. Update October 2016: she has healed and passed the functional hearing tests for the first time in 5 years. She will not need a third set of permanent tubes		Started failing traditional tests at age 5 even after tubes—auditory brainstem response (ABR) performed—normal. Asymmetric, better on the right ear ABR performed with MRI at age 9 and it was normal. 2016 her final (second set) ear tubes were removed and will follow-up this to see how she does without them. Repeat ABR is necessary each year.	
**Sleeping**		**–**	
Did or does your child have any “prolonged sleeping problems?”		Yes (severe ages birth to age 5 waking at 1–4 a.m.). Sleeping issues decreased after the age of 5, the parents refused medication	
**Gastrointestinal and feeding**		**–**	
Childhood feeding problems?		Could not latch, bottle was ok, breast milk ok reflux and still regurgitates food back up in mouth and obstipation	
Feeding tube		No	
GERD or reflux		Yes	
Oral movement difficulties		Yes	
Oral drinking liquid problems (thicken liquids)		Yes	
Does not seem to “getfull^”^		Yes	
Aspiration difficulties		Yes	
Is your child overweight?		Yes	
Constipation problems		Yes	
**D**			
**Body and skeletal**		**–**	
Teeth: did teeth come in-early? (baby teeth, including molars approximately at age 1)		NO	
Feet: shape or abnormality—describe all		Short little toes, chubby/puffy, flat	
Feet: (circulation) cold (abnormally cold)		Yes	
Feet: size		Very small	
Hand: shape or abnormality, describe all		Fifth finger clinodactyly tested by X-ray and confirmed by Mayo ClinicGenetics	
Hands: puffy/pudgy		Small and toddler like	
Hands: fifth finger clinodactyly? (pinky finger bends inward at the last joint)		Yes	
Ankles: pronate inward/bow inward			
Heart: congenital heart defect?		At/after birth it took longer for a valve to close. Murmur now not heard at age 9. Checked out ok for cardiology with recent ADNP diagnosis and ultrasound 2015	
**Endocrinology and growth**		**–**	
Growth delays/“short stature” (below 25% percentile)		YES (diagnosed at the age of 3)	
Growth Hormone - LOW?		Growth hormone was low around age 7	
**Others**		**–**	
Has your child had “breath holding” episodes?		Started at 4 weeks old, stopped around 3 years old (ambulatory care needed and coded at hospital)	
Autoimmune: did/does your child get sick often?		At young ages, high fever, RSV, kidney infection, UTI	
Insensitivity to pain/high pain threshold		Yes	
Temperature regulation issues		Yes	
Circulation: does your child get cold hands and feet?		Yes	
Toilet trained? Daytime?		In the process—76% trained	
Toilet trained? Night time?		No	
Hyperphagia? Excessive appetite-obsessed with eating even if not hungry…		Yes	
Abnormal obsession or desires of drinking water		Yes	
E. Selected p.Tyr719* ADNP-mutated children			
ID	8	10	13
cDNA/nucleotide	c.2157 C>A	c.2157 C>A	c.2157 C>G
Protein—pchange	p.Tyr719*	p.Tyr719*	p.Tyr719*
Sex	Male	Female	Male
Birth Year	2012	2014	2008
Gross motor delay	Yes	Yes	Yes
Fine motor delay	Yes	Yes	Yes
Intellectual disability	Yes	Unknown—too young	Yes
Speech delay	Yes	YES	Yes
ASD diagnosis	Yes	unknown—too young	No
Brain abnormality on MRI	No	YES	Yes
Type of brain abnormality	n/a	Widening of ventricles, cerebral atrophy (volume loss), thinning of the corpus callosum	Frontal atrophy and some volume loss all over. Wide frontal and temporal horns. Small/fine corpus callosum

AD shares the same mutation with several other children [e.g., Ref. (24)]. A parent questionnaire of 17 individuals with ADNP p.Tyr719* or a close mutation indicated cognitive (including speech) and motor delays, as well as autistic features and >50% brain abnormalities, which are further investigated. Figure 1C shows representative pictures of three additional ADNP p.Tyr719* children exhibiting potential facial similarities and using standing and walking devices [Table 2 (E)]. The ADNP p.Tyr719* is an integral part of the ADNP-related syndrome encompassing other ADNP mutations (Van Dijck et al., in preparation).

### Treatment Modalities and Future Perspectives

In general, a helmet treatment was implemented for cranial asymmetry [Table 1 (A)].

#### Treatments for Motor Disabilities

Proved to be successful and should be implemented in future cases. In this respect, all children with ADNP mutations show motor delays (see text footnote 3).

The painful doubt whether AD would be wheelchair bound drove the parents to seek help. Physical therapy began at the age of 15 months. She had multitude therapies at home, collaborative clinic, early intervention, developmental playgroups, and private clinic/water therapy. For example, she had physical and occupational therapy, hippotherapy (involving an occupational therapist, a physiotherapist, or a speech and language therapist working with AD and a horse to present challenges and to promote different postural responses) and aquatic therapy. At one point, she was given up to 10 therapies a week. At the age of 2.5, her therapist and the orthotics^4^ tailor made shoes with an inside lift to accommodate her left leg shortness. She then began to wear ankle foot orthosis brace to her knee, covering her entire foot. The physical therapist devised fabric braces with Velcro with metal bars that did not allow her to bend her knee, forcing her to bare weight. Her father has further built a “standing device” made of wood and PVC pipes to aid in her “timed” standing to ensure that she would not incur atrophy in her lower limbs/legs. Following this, parents were advised of gait trainers and other newly engineered products that help children like AD. AD further used a surfboard therapy machine that helped her core balance and many mirrors and began making a connection. Basic standing on her own was her first milestone, followed by the use of a pediatric walker [Table 1 (B), picture].

Walking short distances resulted in skin redness and muscle pain, which were relieved with warm baths and pool therapy. The parents further used a treadmill program (e.g., http://www.taap-project.com/) in physical therapy to strengthen AD and built new pathways and signals to help her motor planning and focus on the repetition of steps. Metronome was a program^5^ that they have also used that proved to be successful. Notably, the physical therapist persisted, despite AD’s severe unknown motor movement disorder and continued to increase the “frequency” of land and water therapies toward success.

She was treated with over 300 h combined physical and aquatic therapies between the ages of 1–5 years. Without a medical billing code for doctors to use, the term “global delay” did not guarantee insurance coverage nor did it describe the medical complexity of AD. This resulted in expenses of over $2,000 per summer during the walk therapies. Currently, AD gets biweekly physical therapies to maintain and further improve her mobility and has begun growth hormone therapy (age 10), aiming to bring her back to the growth chart.

#### Treatment of Speech Disabilities

AD was recently diagnosed with apraxia of speech [Table 1 (E)] and traditional speech therapy may not benefit her as much as focus on oral motor “planning.” She is now receiving this specialized therapy as her evaluation showed she desires to speak, tries and makes approximations, but similar to walking cannot put it together.

The Z-VIBES^6^ oral motor tool has been most successful for the children with ADNP mutation who speak (closed ADNP Syndrome PARENTS GROUP, Facebook). AD has used it as a small child, with no obvious results, but now it seems to be having a significant effect.

Additionally, the Kaufmann kit,^7^ which uses cards to teach children to combine consonants and vowels to form words while controlling for oral-motor difficulty, is also helping AD. As a small child she was not able to understand, but now these cards are used in combination with PROMPT therapy (Prompts for Restructuring Oral Muscular Phonetic Targets^8^). With physical cueing and repetition of the cards, AD now has some approximations for sounds such as “meh,” “na,” and “beh,” for “me,” “no,” and “bye,” respectively.

Over the years, AD’s educational team has indicated that the autism symptoms have become less prevalent. With access to a speaking device (a card system) and multimodal communication system, AD is now flourishing. Her improved communication shows an amazing personality, which is humorous and lively.

#### Face to the Future: Treatment Using Novel Biologically Active ADNP Peptides

From a scientific point of view, we strive to have tailored drugs to treat the ADNP children, early enough to avoid confounding developmental delays. In this respect, we (Gozes) have been developing NAP (NAPVSIPQ) the shortest active snippet of ADNP (1). NAP increases ADNP’s activity at the cellular level in terms of cellular protection (14) as well as synaptic plasticity (14). On the whole animal level, it provides cognitive protection in mice with ADNP deficiencies (5). NAP has also shown protective activity in over 20 cell culture systems and in over 25 animal models. Notable examples include schizophrenia (36–38), Alzheimer’s disease (39–41), frontotemporal dementia (42, 43), amyotrophic lateral sclerosis (44), and most importantly cognitive deficiencies associated with ASD (5) as well as developmental delays linked to prenatal and postnatal toxicities (45–47). NAP, also called davunetide, has shown activity in clinical trials. It increased cognitive scores in a Phase IIa clinical trial in patients suffering from amnestic mild cognitive impairment (48, 49) and protected functional daily activities (50) as well as brain cellular activity (measured by MRI) (51), in schizophrenia patients. NAP (davunetide) is safe and bioavailable (52, 53). Clinical studies in the ADNP-related syndrome are currently planned with NAP—now called CP201 (Coronis Neurosciences^9^).

## Conclusion

The described complex syndrome of motor dysfunction, autism, speech delay, and facial dysmorphism should trigger gene sequencing and early diagnosis of children with the ADNP syndrome (also known as the Helsmoortel–Van der Aa syndrome^10^).

The case of AD highlights global developmental delays, with emphasis on motor functions, which was not described before. While AD had not always been referred to the appropriate specialized therapies, her family persistence and ultimate success in motor development should help guide newly diagnosed children. This report teaches modes of interventions and paves the path to basic discoveries on ADNP’s involvement in muscle development.

AD’s major impediment nowadays is her inability to talk, a trait that has been lagging behind. In this respect, better understanding of ADNP’s association with muscle development may shed light on speech development as well.

The more we understand about the ADNP protein and gene, the better we can help the families and patients with syndromic developmental dysfunctions gain enhanced functionality and integration into a healthy society coupled with ADNP replacement/enhancement therapies, such as CP201 (Coronis Neurosciences; see text footnote 9).

## Ethics Statement

This study was supported by Tel Aviv University, the study is descriptive the parent is an author. Informed consents were given for all data by the respective parents.

## Author Notes

Recently (at about age 11) AD was diagnosed with a suspected form of dysarthria—https://www.ncbi.nlm.nih.gov/pubmedhealth/PMHT0027115/. The number of ADNP syndrome children has now increased to 107 within the ADNP network (http://www.adnpfoundation.org/), including 23 children sharing a similar mutation with AD (Table 2).

## Author Contributions

IG compiled the data and wrote/edited the paper; MP provided the in-depth clinical analysis at 3 years of age and the longitudinal MRI data; AVD and FK read the manuscript and provided clinical input; JP provided the diagnosis and longitudinal clinical data, JE provided critical editorial inputs; AZ-D initiated the study and provided the most extensive parental story, and SB-S compiled the data in Table 2, read, and commented on the paper.

## Conflict of Interest Statement

Coronis Neurosciences is developing NAP (davunetide, CP201) for autism. Professor Gozes is the Chief Scientific Officer (Consultant).

## Footnotes

^1^https://www.cdc.gov/growthcharts/data/set1clinical/cj41c022.pdf.

^2^http://www.omim.org/entry/611386.

^3^http://www.adnpkids.com/.

^4^https://en.wikipedia.org/wiki/Orthotics.

^5^https://www.youtube.com/watch?v=WR1lC6UYh9I.

^6^http://www.arktherapeutic.com/z-vibes-all/.

^7^http://www.northernspeech.com/early-intervention-language-and-speech/kaufman-k-slp-treatment-kit-1-ndash-basic-level/.

^8^http://www.promptinstitute.com/?page=FamiliesWIP.

^9^http://www.coronisns.com/.

^10^https://rarediseases.info.nih.gov/diseases/12931/adnp-syndrome.
